# Supplementary material for: Structure of Rap1b bound to talin reveals a pathway for triggering integrin activation
Source: Nat Commun. 2017 Nov 23;8:1744. doi: 10.1038/s41467-017-01822-8 (PMC5701058; doi:10.1038/s41467-017-01822-8)
Supplement: Supplementary file 1 — Supplementary Information [file 41467_2017_1822_MOESM1_ESM.pdf]

Supplementary Figure 1

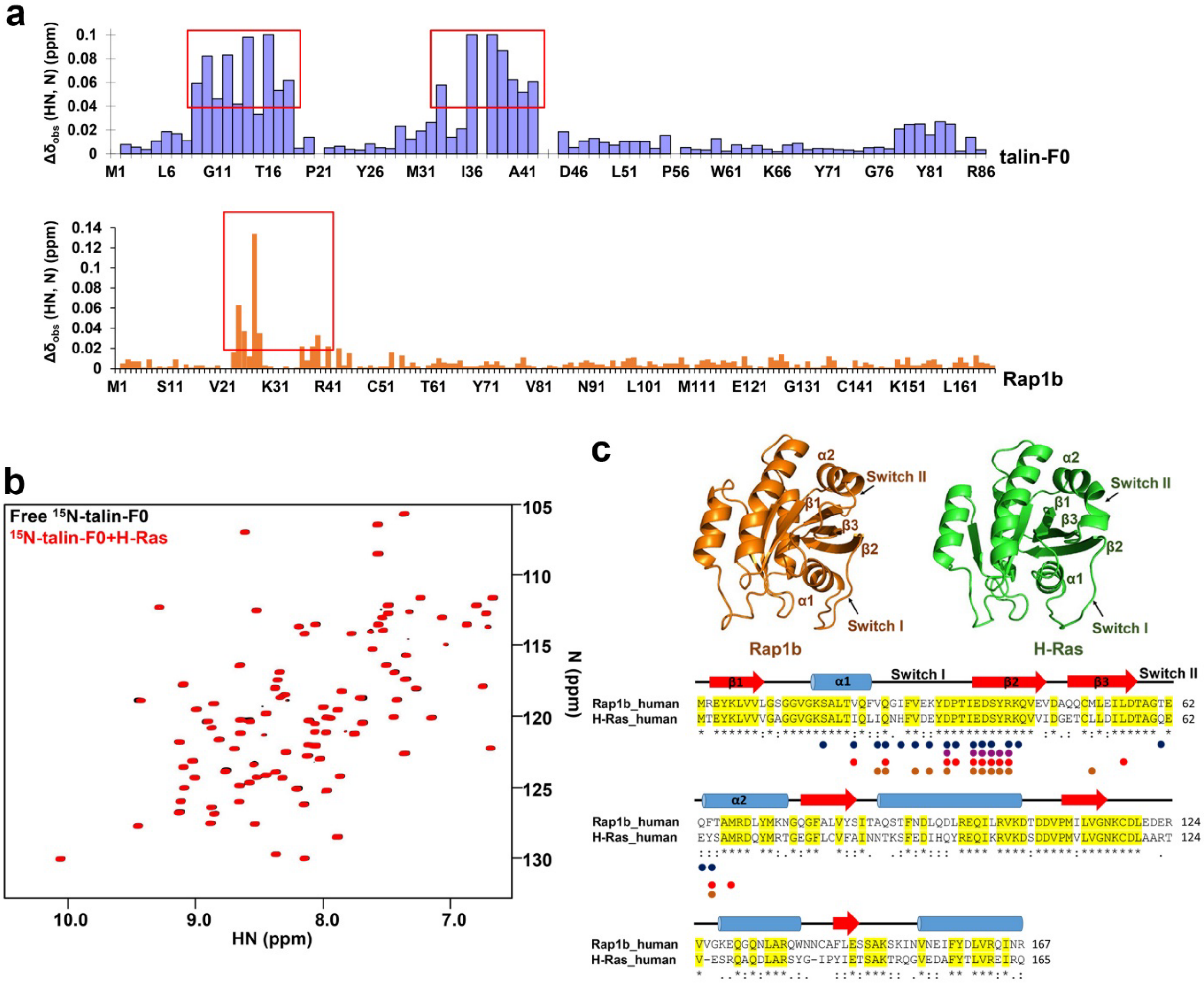

Supplementary Figure 1. Rap1b but not H-Ras interacts with talin-F0 (**a**) Upper panel, the profile of chemical shift changes of 50 μM ^{15}N-talin-F0 induced by 125 μM GMP-PNP loaded Rap1b. The chemical shift changes of completely broadened residues (T16, I36 and E38) were set to 0.1 ppm. The most perturbed regions are boxed in red. Lower panel, the profile of chemical shift changes of 45 μM GMP-PNP loaded ^{15}N-labeled Rap1b (1-167) induced by 225 μM talin-F0. The most perturbed region is boxed in red. (**b**) The HSQC spectra of 50 μM ^{15}N-labeled talin-F0 in the absence (black) and presence of 125 μM GMP-PNP loaded H-Ras (red). (**c**) Sequence alignment of Rap1b and H-Ras. Identical residues are highlighted in yellow. ('*' indicates fully conserved residue, ':' indicates residues with strongly similar properties, and '.' indicates residues with weakly similar properties. Residues of Rap1b involved in the binding interface (cut-off of 4 Å) with talin-F0, RIAM-RA, KRIT1-F1, and c-Raf1-RA are indicated in the figure with blue, purple, red and brown solid circles respectively.

Supplementary Figure 2

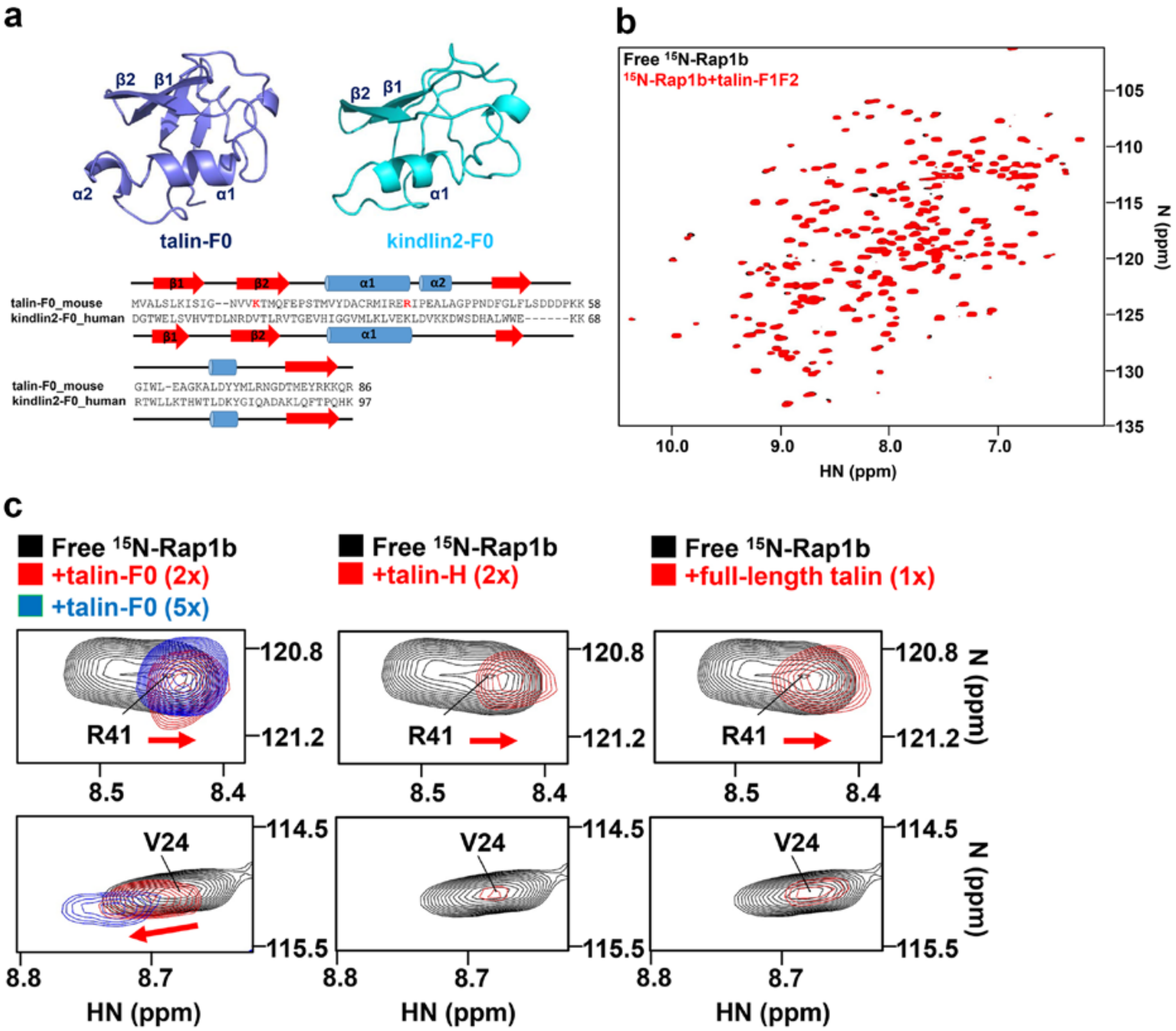

Supplementary Figure 2. Neither talin-F1F2 nor kindlin2-F0 interacts with Rap1b (**a**) Structure-based sequence alignment of talin-F0 and kindlin2-F0. (**b**) The HSQC spectra of 45 µM GMP-PNP loaded ^{15}N-labeled Rap1b (1-167) in the absence (black) and presence of 90 µM talin-F1F2 (red). (**c**) The HSQC spectra (representative regions were shown) of 45 µM GMP-PNP loaded ^{15}N-labeled Rap1b (1-167) in the absence (black) and presence of 90 µM talin-F0 (red), 225 µM talin-F0 (blue), 90 µM talin-H (red), or 45 µM full-length talin (red). Note that the peak of V24 was broadened in the presence of talin-H or full-length talin.

Supplementary Figure 3

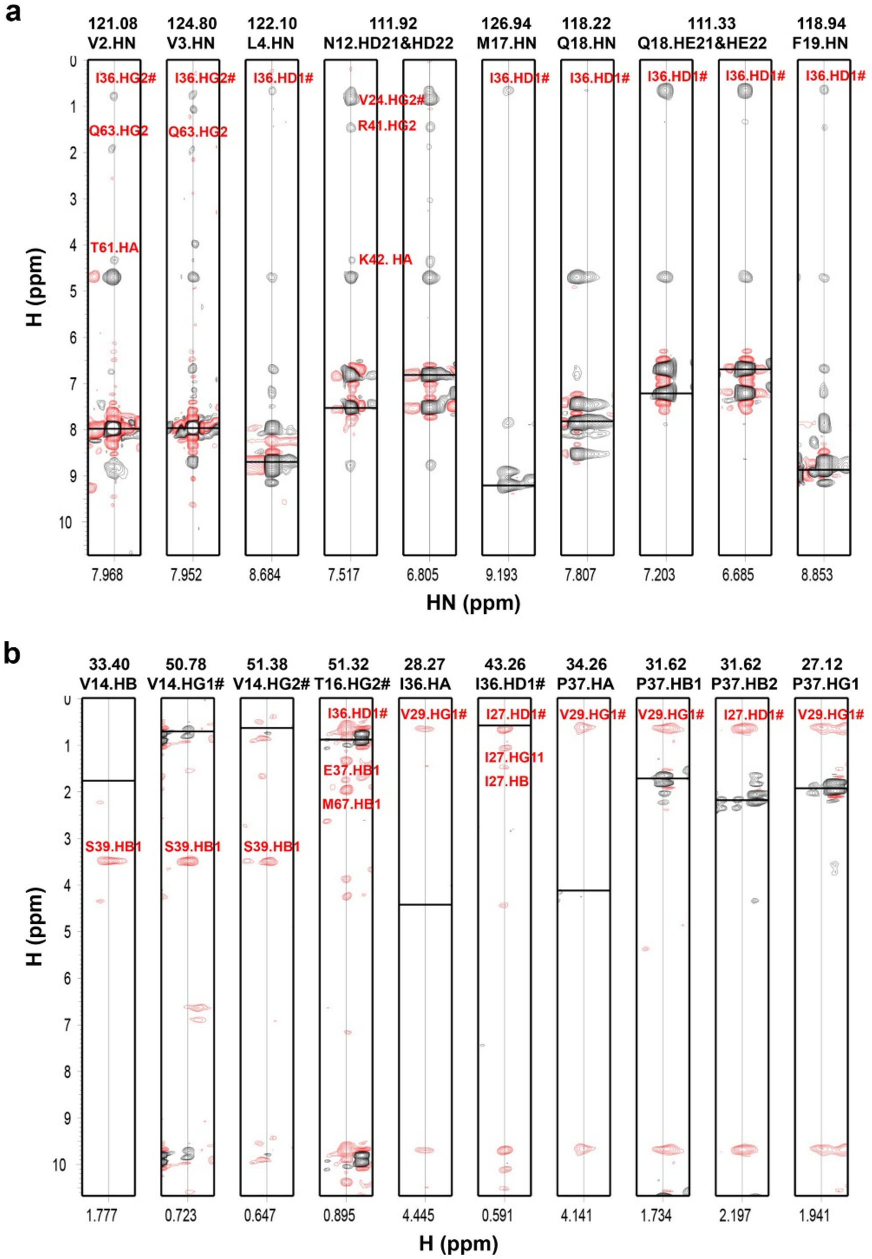

Supplementary Figure 3. Representative intermolecular NOEs for the structure calculation of Rap1b/talin-F0 complex (**a**) Representative intermolecular NOEs obtained from 3D ^{15}N-edited NOESY experiment (300 ms mixing time) with sample 3: 0.5 mM ^{15}N/100% ^{2}H-labeled talin-F0 in the presence of 0.7 mM unlabeled Rap1b. Selected residues of talin-F0 were indicated on top of each strip. (**b**) Representative intermolecular NOEs obtained from 3D ^{15}N/^{13}C-filtered NOESY experiment (120 ms mixing time) with sample 4: 0.5 mM ^{15}N/^{13}C-labeled talin-F0 in the presence of 0.7 mM unlabeled Rap1b prepared in 99.8% D_2O. Selected residues of talin-F0 were indicated on top of each strip. Cross-peaks shown in both (a) and (b) which were unambiguously assigned to the specific proton of unlabeled Rap1b residues are labeled in red. The horizontal black line of each strip is diagonal line whose position indicates the chemical shift of the specific proton labeled on top of each strip.

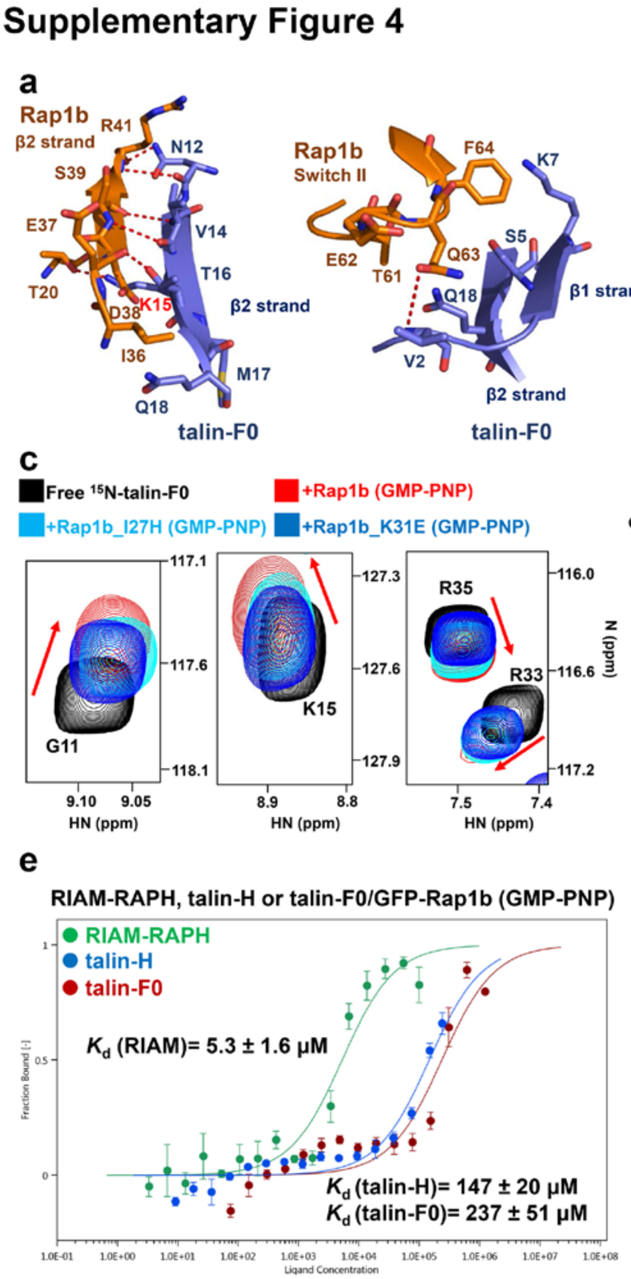

Supplementary Figure 4. Rap1b/talin-F0 interaction in the absence of membrane is modest but highly specific (**a**) Detailed binding interface between β2 strand or switch II region of Rap1b and talin-F0. Hydrogen bonds are represented in red dashed lines. (**b**) A distinct hydrophobic core formed between I36/P37 (shown in spheres representation) of talin-F0 and V21/I27/V29 of Rap1b (shown in surface representation). (**c**) The HSQC spectra (four representative residues were shown) of 50 μM ^{15}N-labeled talin-F0 in the absence (black) and presence of 125 μM GMP-PNP loaded Rap1b (red), K31E mutant (blue) or I27H mutant (cyan). Note that all Rap1b variants also bear a G12V mutation. K31E and I27H mutations resulted in overall less chemical shift changes and less extent of line broadening. (**d**) GST pull down assay to show that talin-H interacts specifically with Rap1b while RIAM interacts with both Rap1b and H-Ras equally. Full blot/gel images are shown in **Supplementary Fig. 16.** (**e**) The affinity between GMP-PNP loaded GFP-Rap1b and RIAM-RAPH, talin-F0 or talin-H measured by Nanotemper. Experiments were done in triplicates. The affinity of GFP-Rap1b/talin-H shown here is an estimated value because the saturation step was not reached due to the aggregation issue of talin-H at high concentration.

Supplementary Figure 5

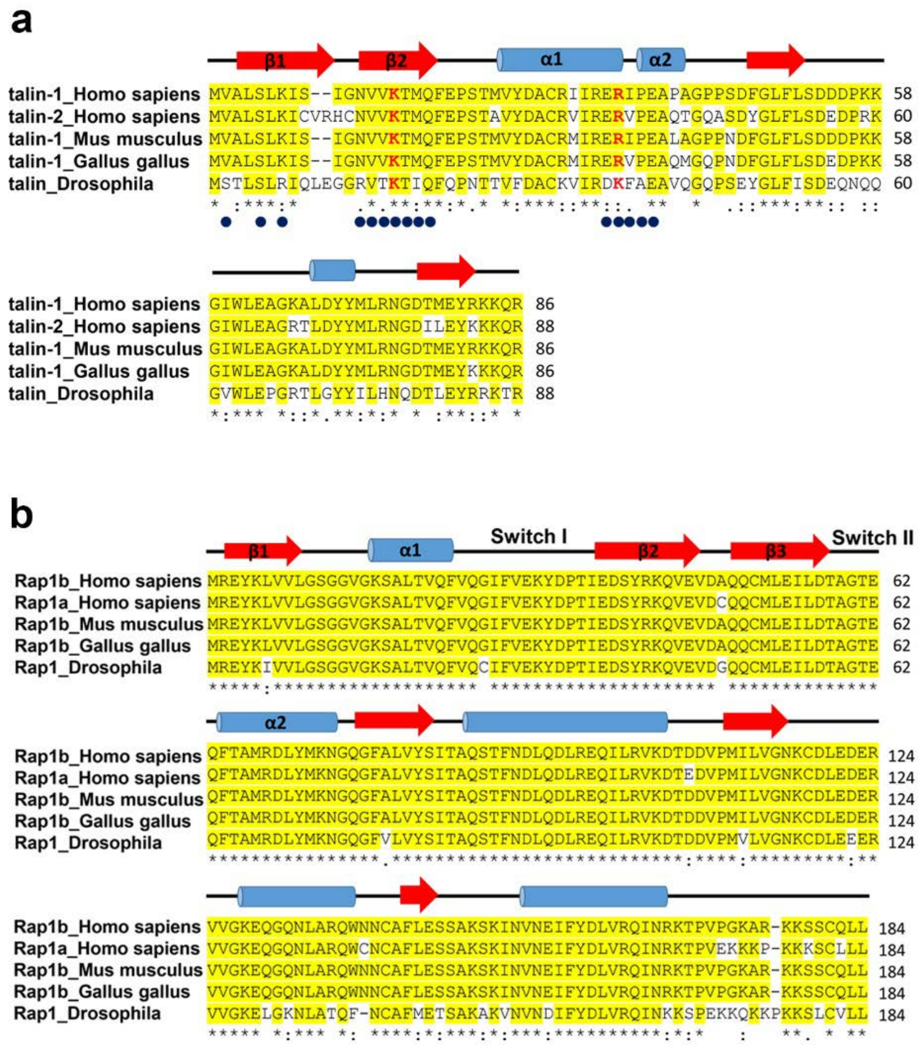

Supplementary Figure 5. Rap1/talin interaction is evolutionally conserved (**a**) Sequence alignment of talin-F0 from different species. Residues of talin-F0 involved in the binding interface (cut-off of 4 Å) with Rap1b are indicated in the figure with blue solid circles. (**b**) Sequence alignment of Rap1 from different species. (Residues identical to human talin-1 or Rap1b are highlighted in yellow. '*' indicates fully conserved residue, ':' indicates residues with strongly similar properties, and '.' indicates residues with weakly similar properties).

Supplementary Figure 6

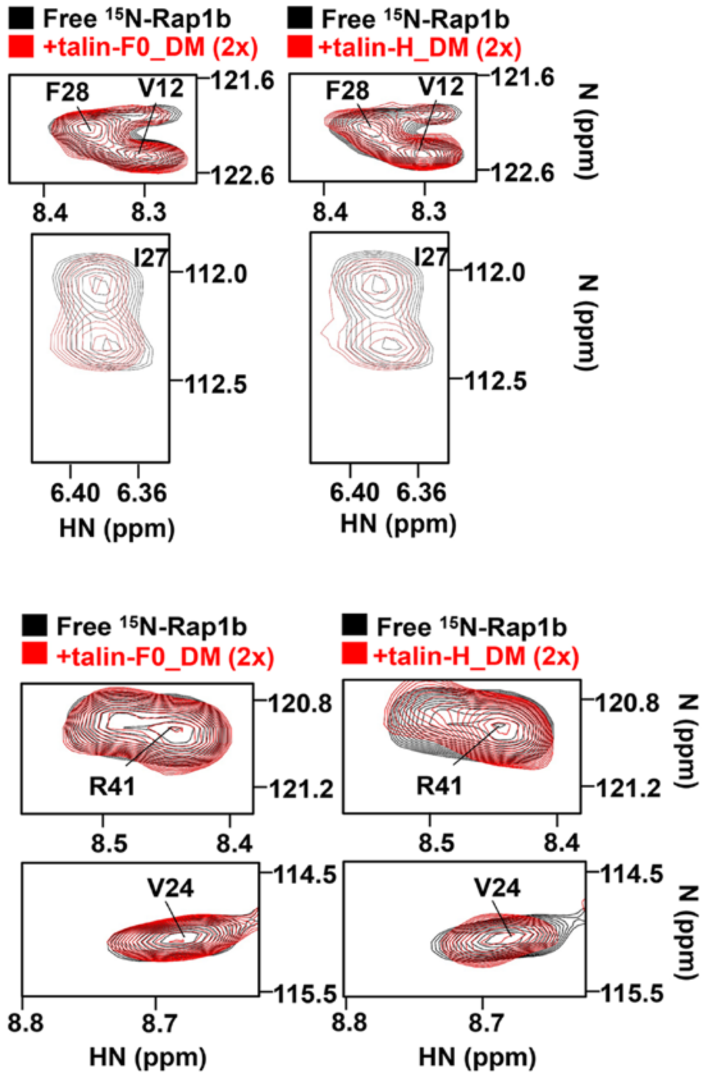

Supplementary Figure 6. Rap1b/talin interaction is drastically reduced by double mutations (K15A, R35A) in talin-F0 domain The HSQC spectra (representative regions were shown) of 45 μM GMP-PNP loaded ^{15}N-labeled Rap1b (1-167) in the absence (black) and presence of 90 μM talin-F0_DM or talin-H_DM (red).

Supplementary Figure 7

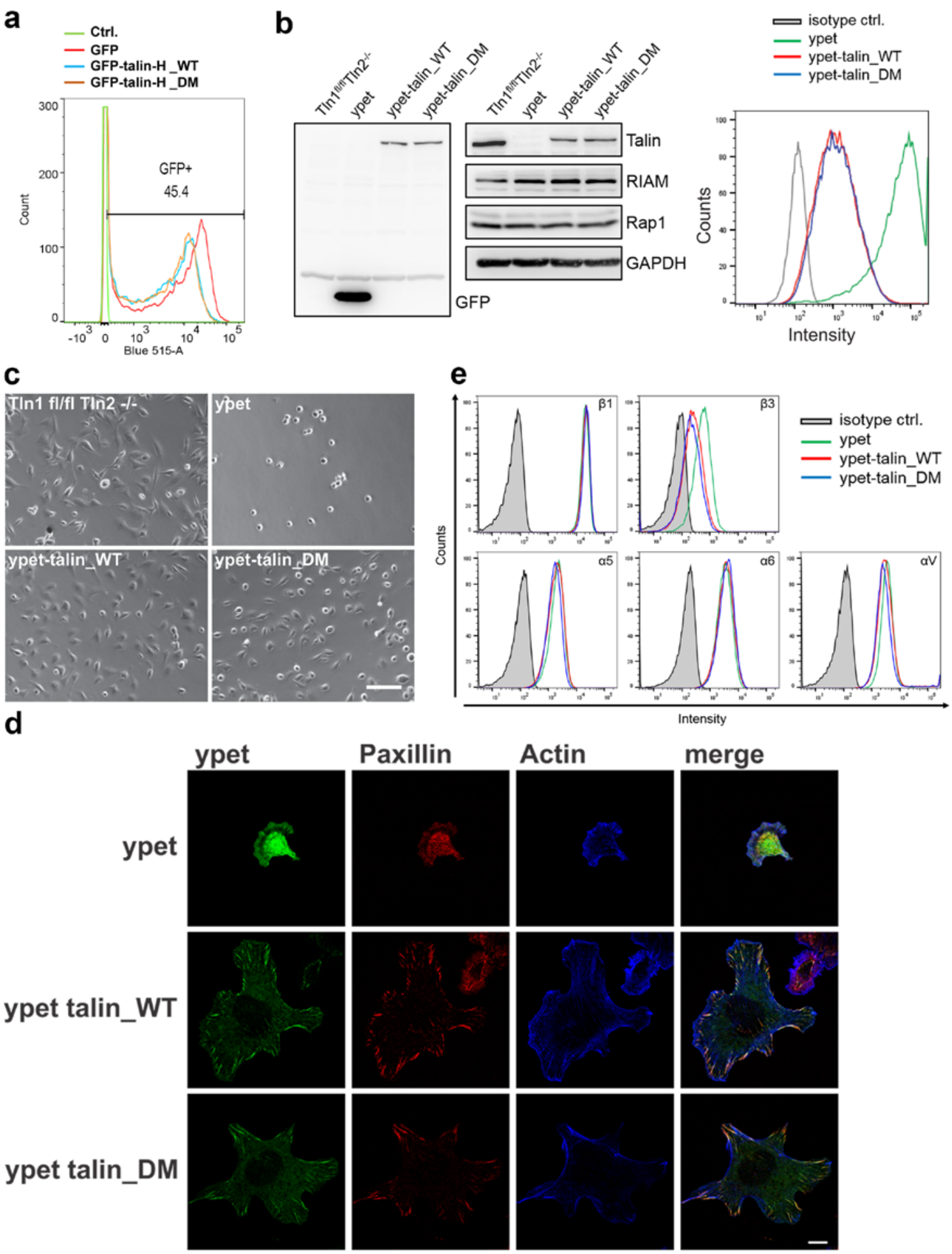

Supplementary Figure 7. Impaired Rap1/talin interaction results in defective integrin activation (**a**) Expression levels of GFP (red), GFP-tagged talin-H WT (cyan) and GFP-tagged talin-H double mutant (DM) (orange) in transfected CHO A5 cells analyzed by flow cytometry. (**b**) Left, western blot analyses of talin control (talin-1$^{fl/fl}$/talin-2$^{-/-}$) and talin$^{1/2dko}$ fibroblasts which were retrovirally transduced with either ypet alone, ypet-tagged

talin WT or ypet-tagged talin DM (K15A, R35A) to show expression levels of ypet and ypet-tagged talin (recognized by GFP antibody), talin, RIAM and Rap1. GAPDH served as loading control. Right, flow cytometric analysis of talin control (talin-1$^{fl/fl}$/talin-2$^{-/-}$) (grey) and talin$^{1/2dko}$ fibroblasts retrovirally transduced with ypet alone (green), ypet-tagged talin WT (red) or ypet-tagged talin DM (blue) to quantify expression levels of the transduced proteins. Full blots are shown in **Supplementary Fig. 17**. (**c**) Phase contrast images of talin-1$^{fl/fl}$/talin-2$^{-/-}$ control cells and talin$^{1/2dko}$ cells expressing ypet, ypet-talin WT or ypet-talin DM plated on fibronectin. Scale bar 100μm. (**d**) Confocal images of the ventral side of talin$^{1/2dko}$, ypet-talin WT or ypet-talin DM cells stained for paxillin (red) and F-actin (blue). Ypet-signal is shown in green. Scale bar 10μm. (**e**) Surface expression levels of integrin β1, β3, α5, α6 and αV on talin control (grey) and talin$^{1/2dko}$ fibroblasts expressing ypet (green), ypet-tagged talin WT (red) or ypet-tagged talin double mutant (DM) (blue) analyzed by flow cytometry.

Supplementary Figure 8

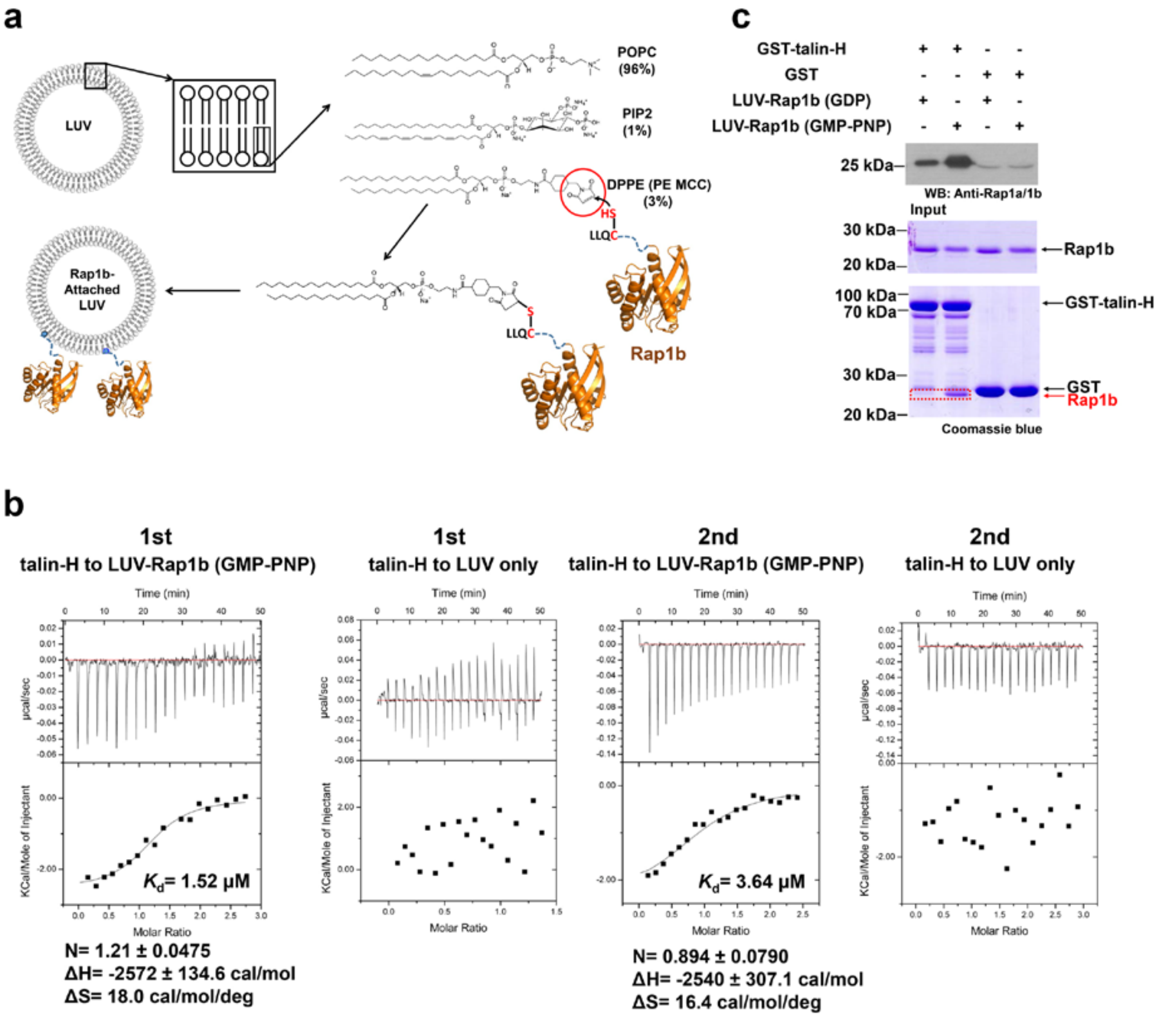

Supplementary Figure 8. Membrane anchored Rap1b robustly enhances its binding to talin (**a)** The diagram procedure of generating Rap1b-anchored LUVs. (**b**) Binding affinity between membrane-anchored Rap1b and talin-H measured by isothermal titration calorimetry (ITC). The titrations of talin-H to LUV-only showed different background heats due to different batches of LUVs, but the enthalpy changes and affinities of talin-H/LUV-Rap1b were consistent after subtraction. N, the number of binding sites. ΔH, enthalpy change. ΔS, entropy change. (**c**) GST pull down assay to show that the interaction between membrane-anchored Rap1b and talin-H is also in a GTP dependent manner. **Full blot/gel images are shown in Supplementary Fig. 18.**

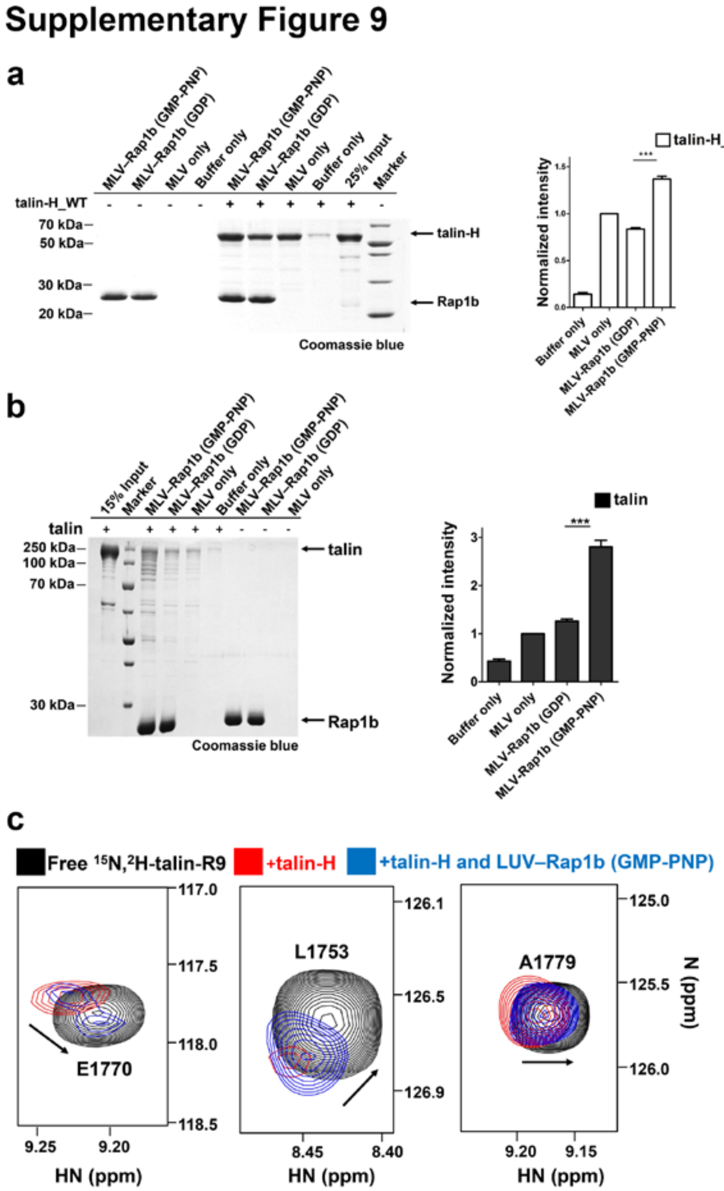

Supplementary Figure 9. Membrane anchored Rap1b interacts with talin in a GTP dependent manner and promotes talin unmasking (**a**) Left, a representative vesicle co-sedimentation assay showing that the interaction between talin-H and membrane-anchored Rap1b is GTP dependent. Right, the quantification of four independent experiments. The intensity of talin-H_WT band was normalized to that of "MLV only" group and the data are shown in means ± S.E.M..*** denotes p <0.0001. Full gel image is shown in **Supplementary Fig. 19**. (**b**) Left, a representative vesicle co-sedimentation assay showing that the interaction between full-length talin and membrane-anchored Rap1b is GTP dependent. Right, the quantification of four independent experiments. The intensity of talin band was normalized to that of "MLV only" group and the data are shown as means ± S.E.M..*** denotes p<0.0001. Full gel image is shown in **Supplementary Fig. 19**. (**c**) HSQC-based spectral changes (three representative residues were shown) of 30 μM ^{15}N/80% ^{2}H-labeled talin-R9 in the absence (black) and presence of 22 μM talin-H (red) showing the talin-H masking by the autoinhibitory talin-R9 as reported before (Song et al., 2012). Addition of 11 μM membrane-anchored Rap1b shifts the peaks towards the free form of talin-R9 (blue) indicating the talin unmasking. Note that the concentrations of the proteins were kept low, which cause less pronounced chemical shift changes but to avoid precipitation. The peak intensity for each dataset was set to the same scale. These experiments were performed at 28.5 ℃ on Bruker 900 MHz NMR spectrometer. The buffer contained 25 mM NaH_2PO4/Na_2HPO4 (pH 6.8), 100 mM NaCl, 5 mM $MgCl_2$, 5 mM β-mercaptoethanol (β-ME) and 5% D_2O.

Supplementary Figure 10

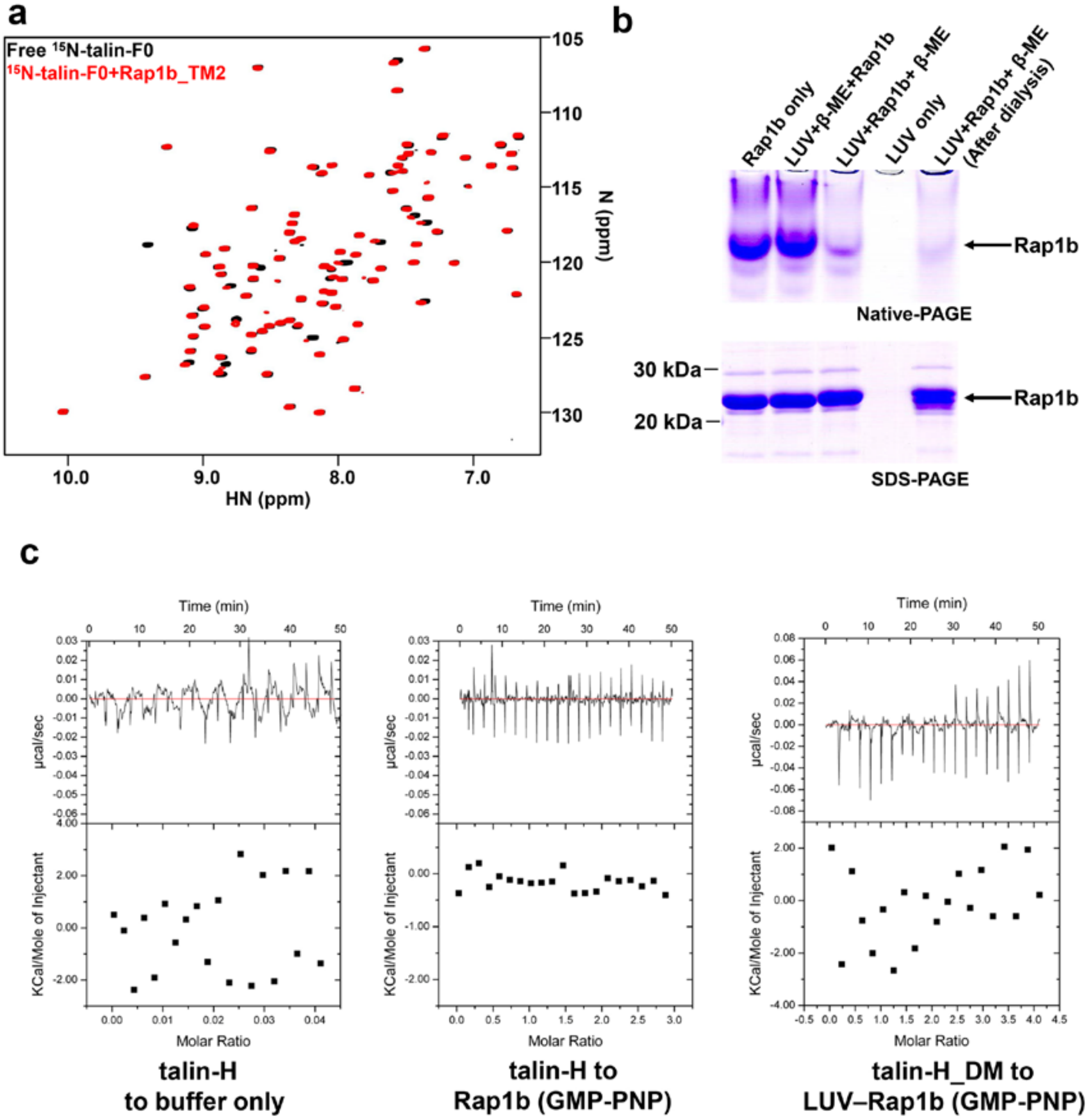

Supplementary Figure 10. The anchorage of Rap1b to membrane vesicles and isothermal titration calorimetry (ITC) control experiments (**a**) The HSQC spectra of 45 μM ^{15}N-labeled talin-F0 in the absence (black) and presence of 90 μM GMP-PNP loaded Rap1b_TM2 (red). (**b**) A combination of Native-PAGE and SDS-PAGE analysis to confirm that Rap1b was anchored to large unilamellar vesicles (LUVs) efficiently. Upper panel, free un-denatured Rap1b migrated into a native-gel, but LUVs anchored Rap1b couldn't due to the increasing size of the whole molecule, shown as "LUV+Rap1b+β-ME" in which the intensity of Rap1b band was significantly reduced. As a control, LUVs pre-incubated with β-ME failed to anchor Rap1b (shown as "LUV+β-ME+Rap1b") and the intensity of Rap1b band remained unchanged. Lower panel, SDS-PAGE to confirm that the total amount of Rap1b remained the same. Full gel images are shown in **Supplementary Fig.18.** (**c**) ITC control experiments as indicated in the figure.

Supplementary Figure 11

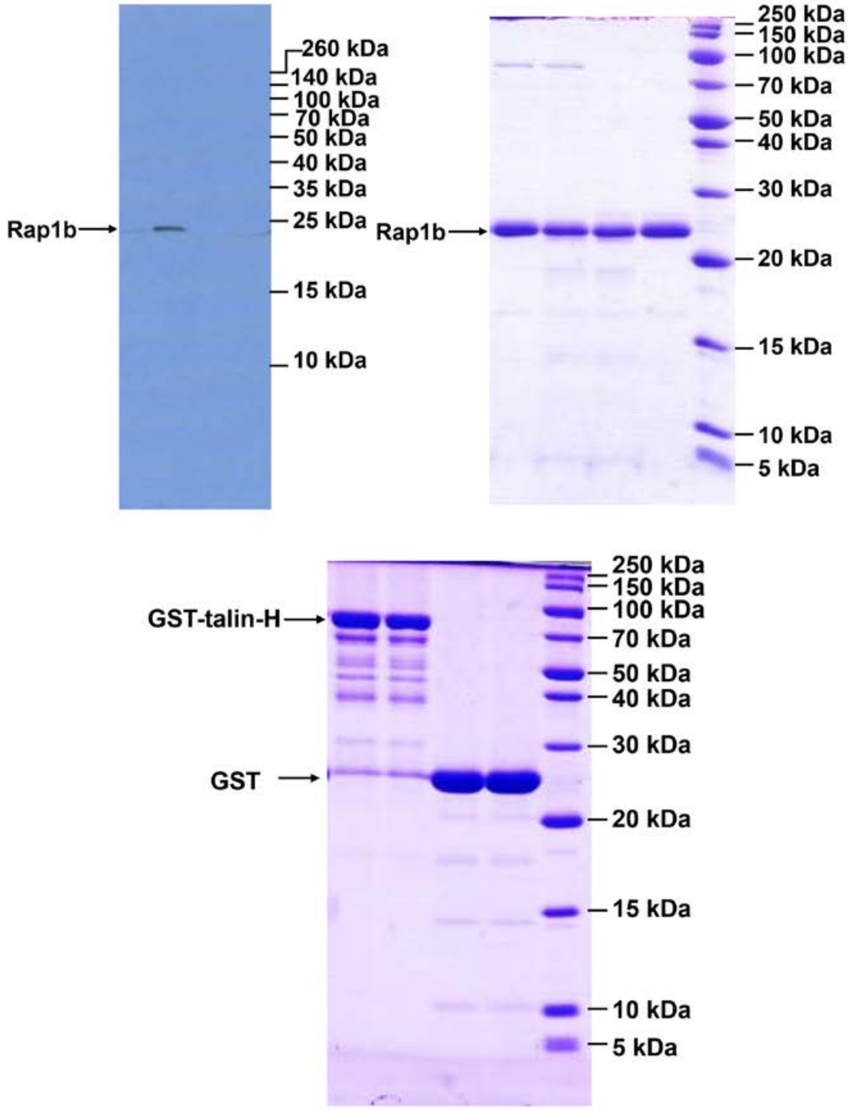

Supplementary Figure 11. Full blot/gel images for Fig. 3b (left panel)

Supplementary Figure 12

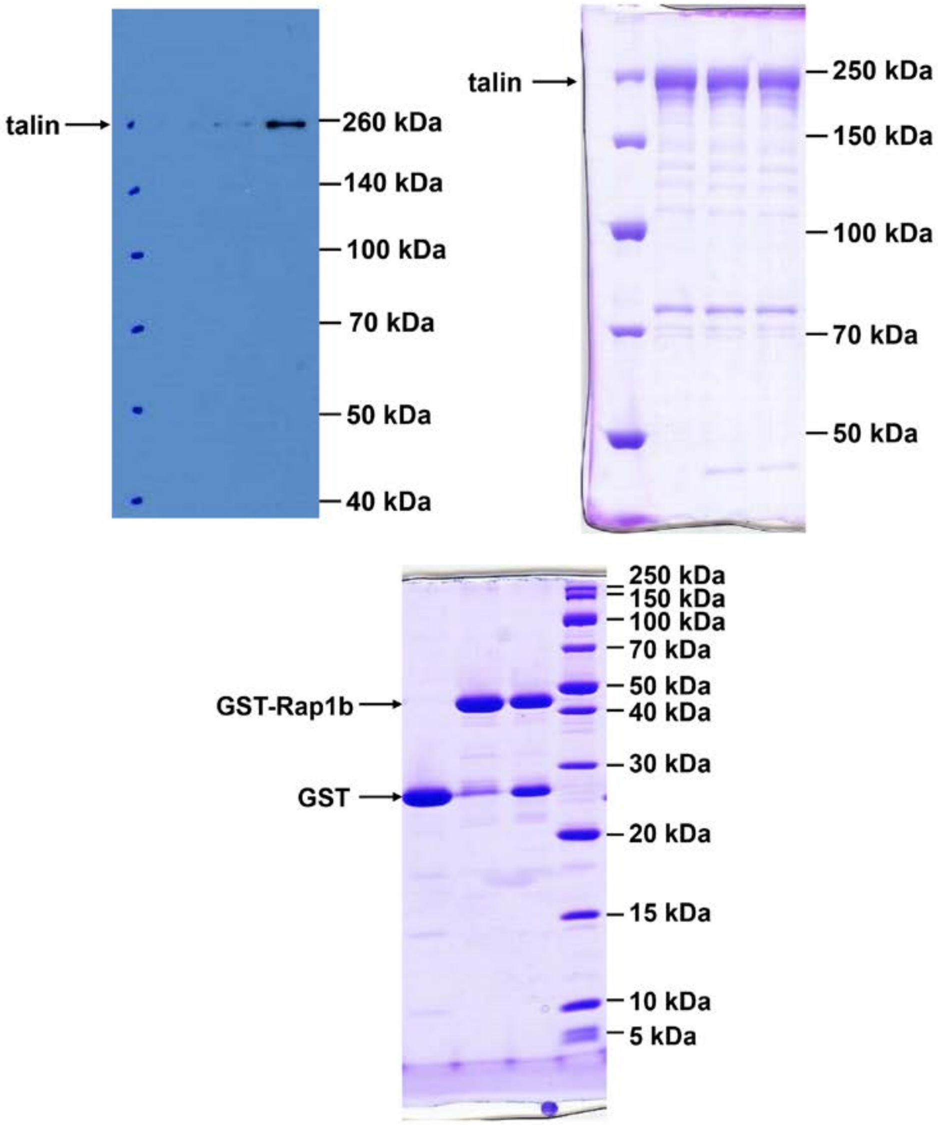

Supplementary Figure 12. Full blot/gel images for Fig. 3b (right panel)

Supplementary Figure 13

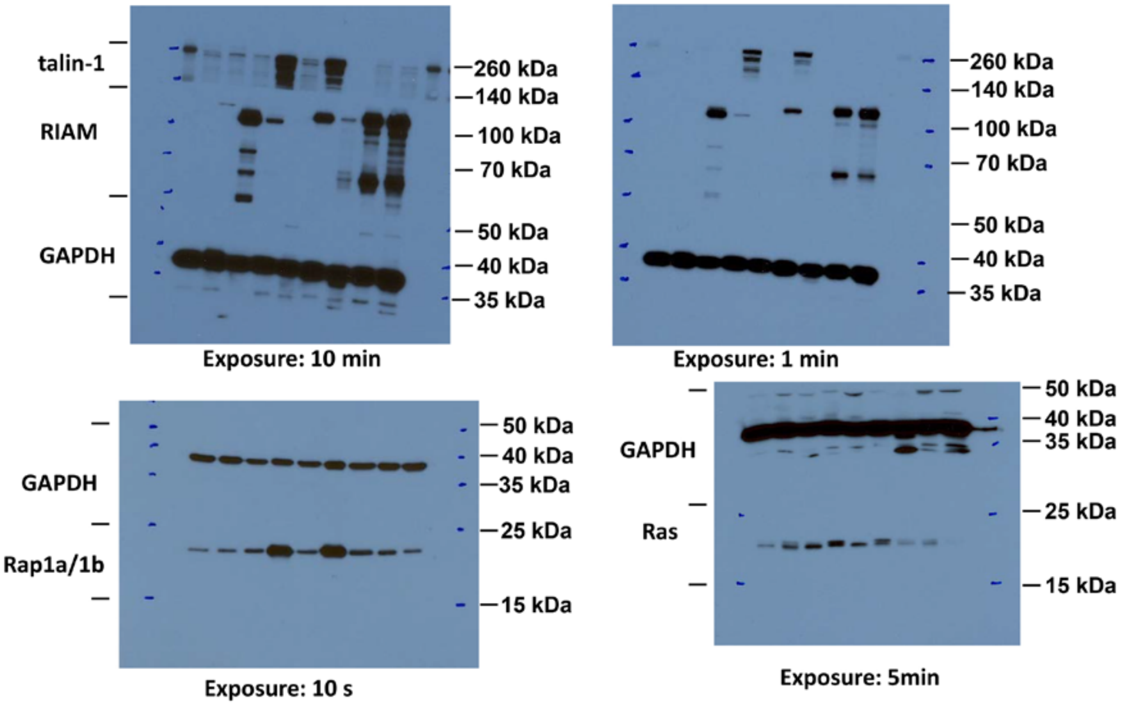

Supplementary Figure 13. Full western-blot images for Fig. 6a

Supplementary Figure 14

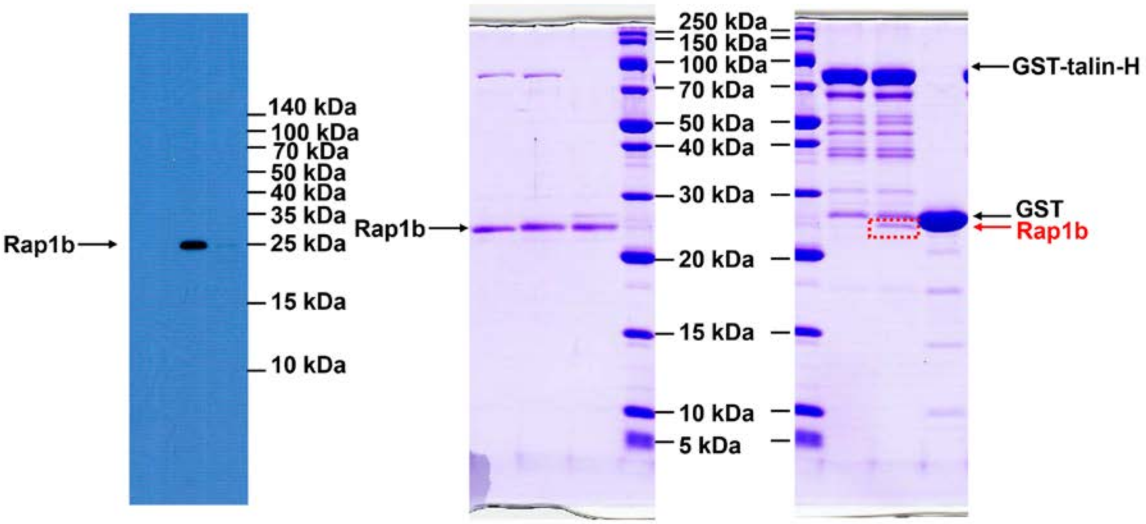

Supplementary Figure 14. Full blot/gel images for Fig. 6c

Supplementary Figure 15

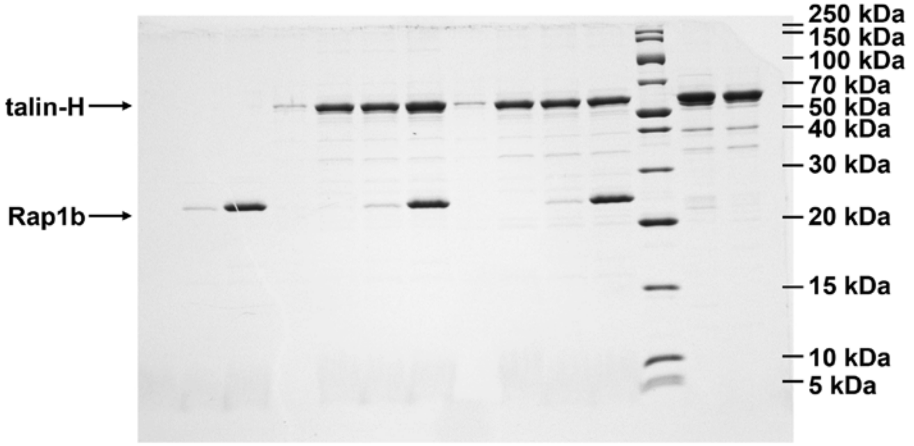

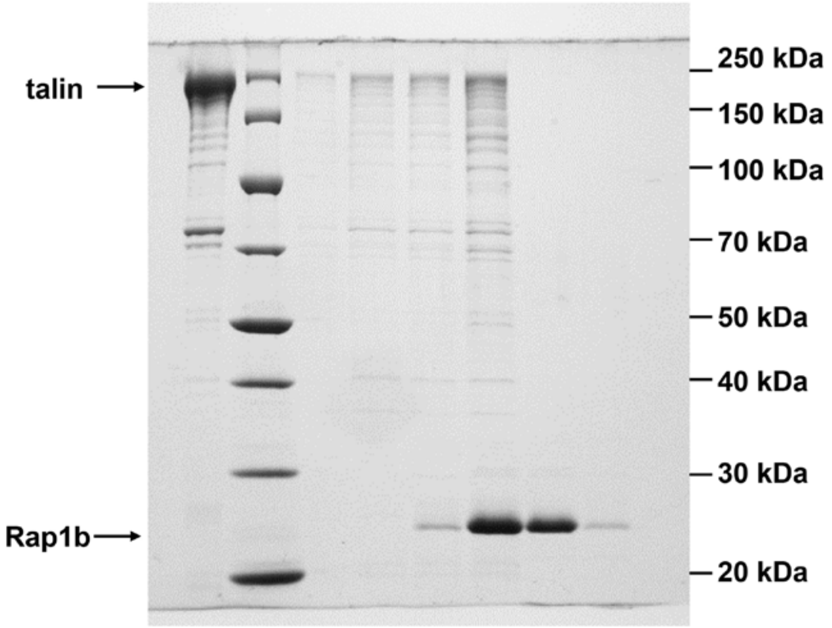

Supplementary Figure 15. Full gel images for Fig. 6d, e

Supplementary Figure 16

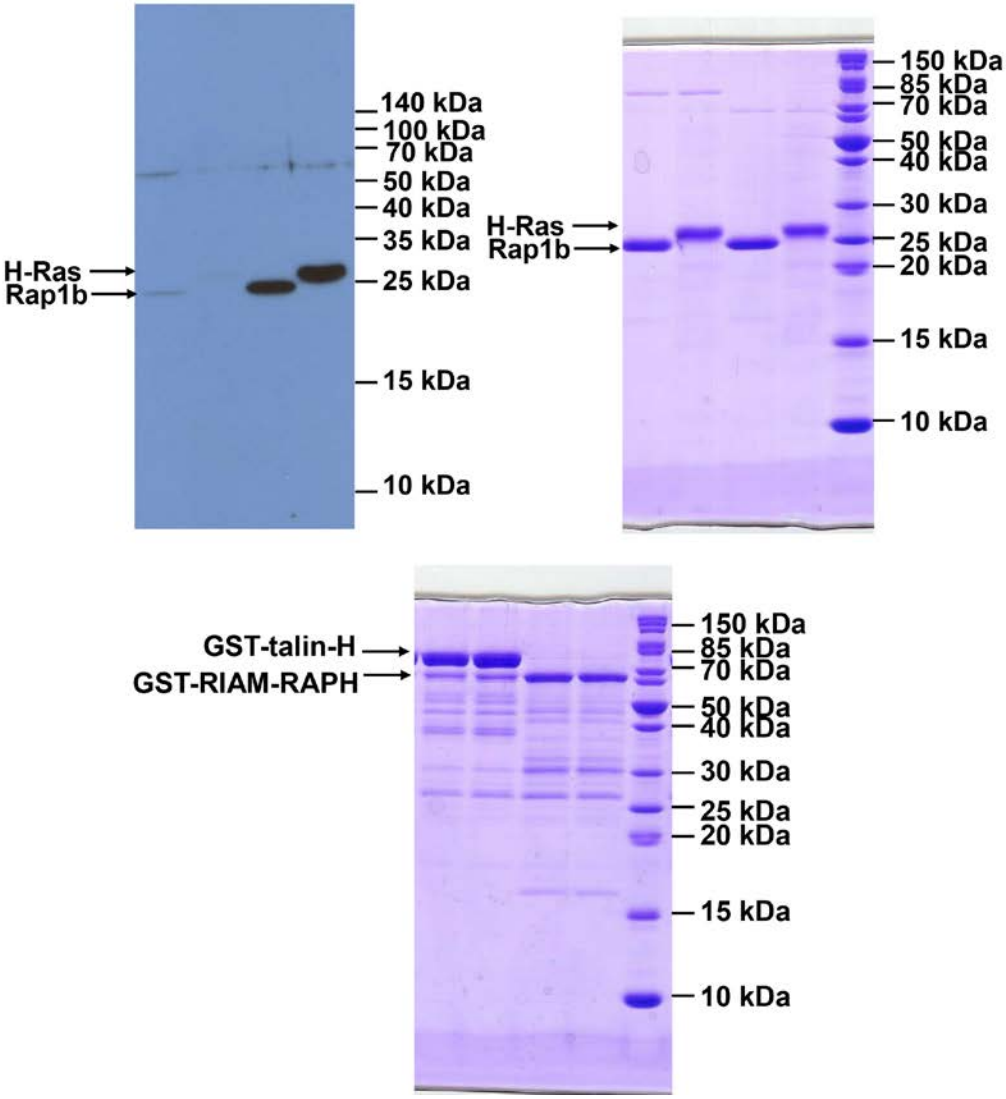

Supplementary Figure 16. Full blot/gel images for Supplementary Fig. 4d

Supplementary Figure 17

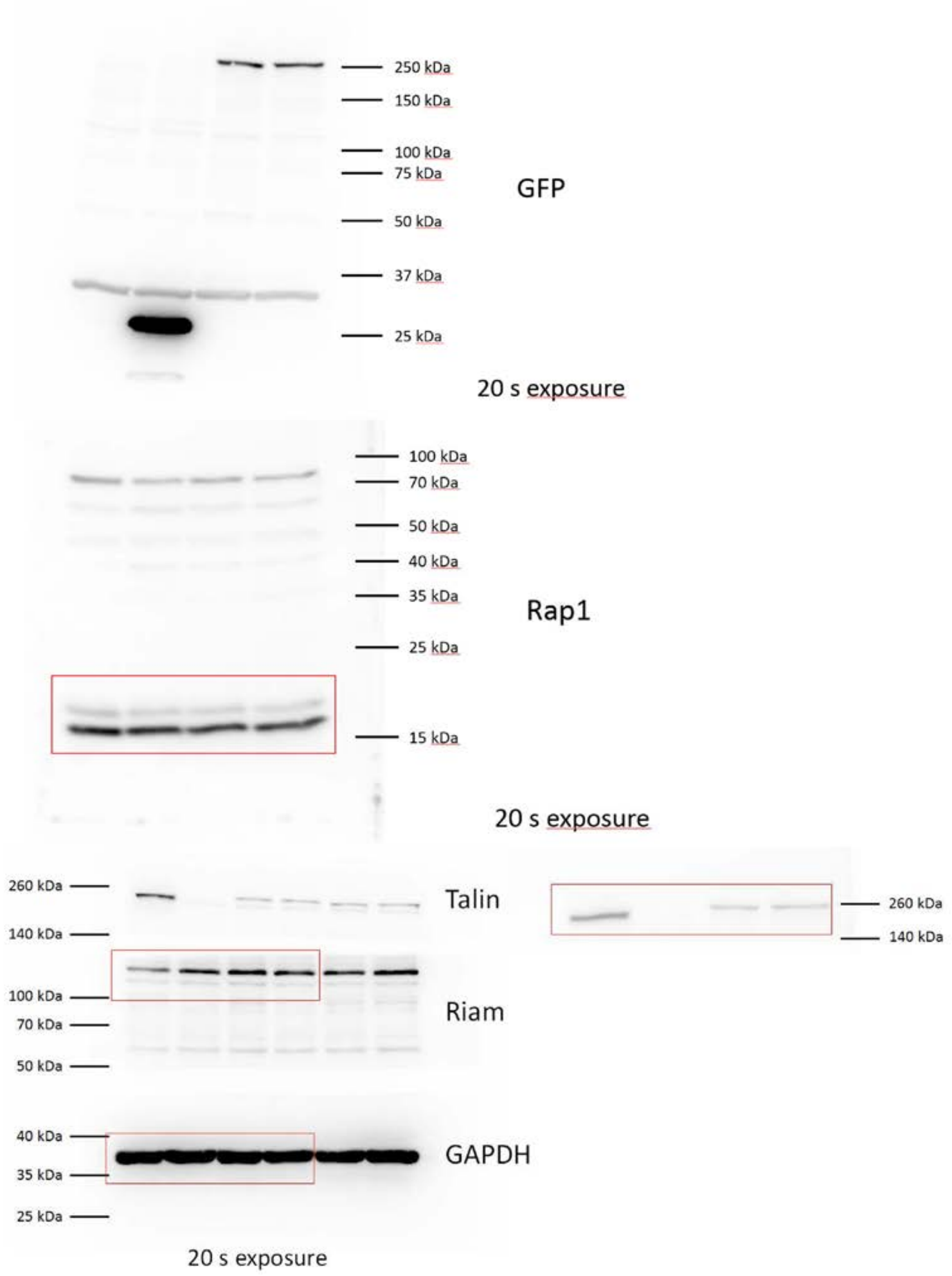

Supplementary Figure 17. Full blot/gel images for Supplementary Fig. 7b

Supplementary Figure 18

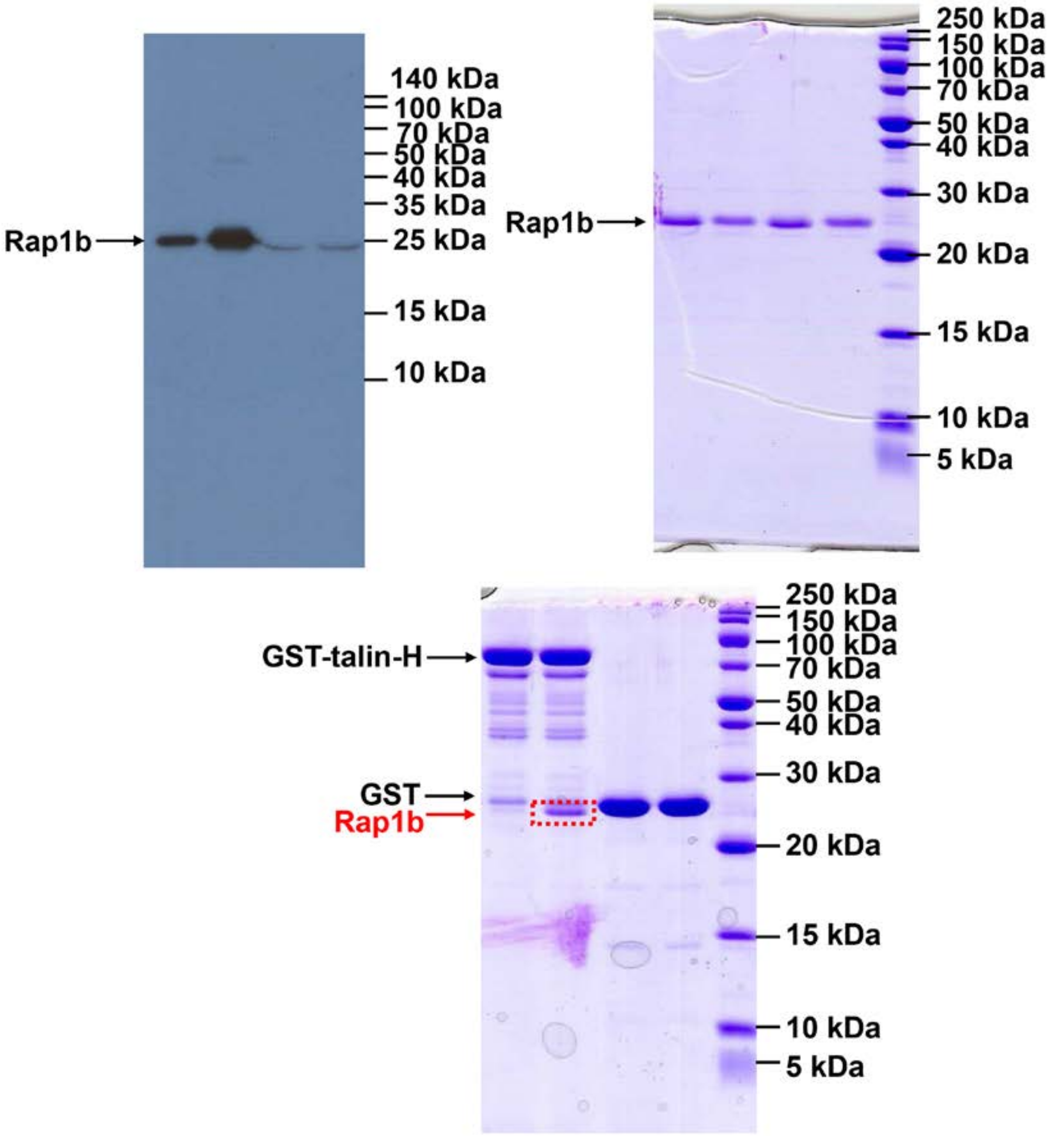

Supplementary Figure 18. Full blot/gel images for Supplementary Fig. 8c

Supplementary Figure 19

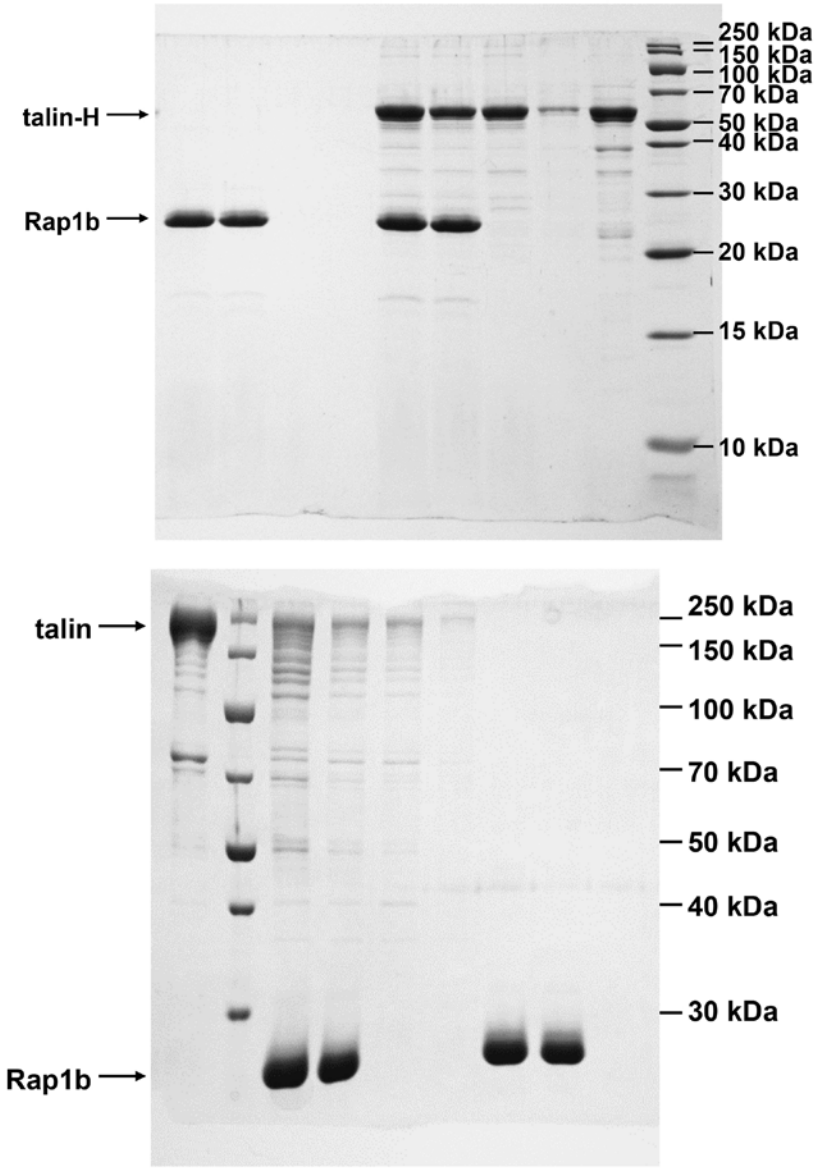

Supplementary Figure 19. Full gel images for Supplementary Fig. 9a, b

Supplementary Figure 20

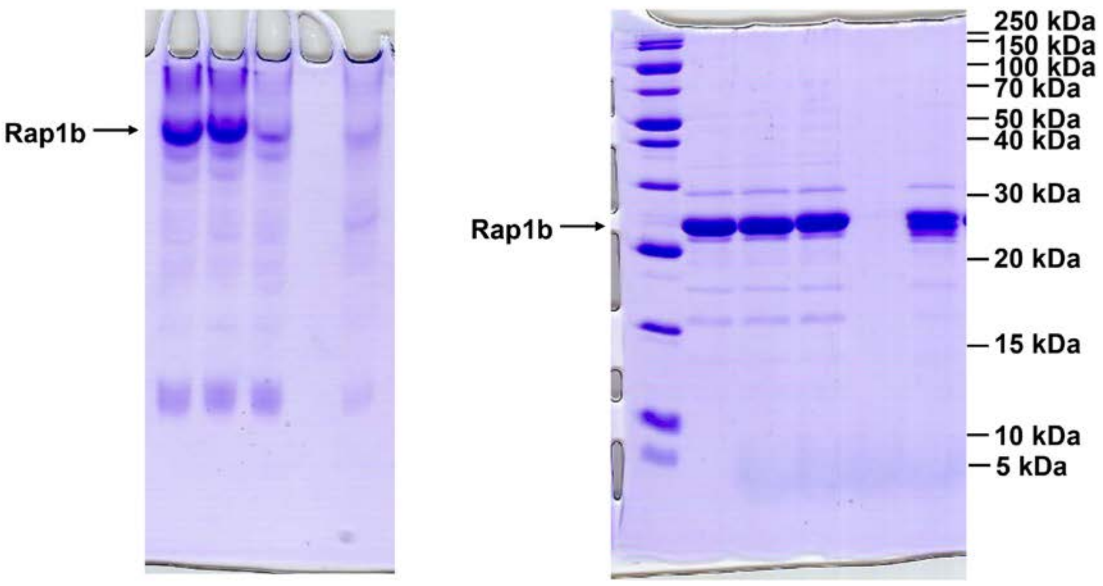

Supplementary Figure 20. Full gel images for Supplementary Fig. 10b

Supplementary Table 1. Structural Statistics of the Rap1b/talin-F0 complex

	Complex
**NMR distance & dihedral constraints**	3802
Distance constraints	3500
Total NOE	3500
Intra-residue	894
Inter-residue	
Sequential (|i-j| = 1)	1158
Medium-range (|i-j| < 5)	620
Long-range (|i-j| ≥ 5)	750
Intermolecular	78
Hydrogen bonds[a]	
Total dihedral angel restraints	302
phi	151
psi	151
Structure Statistics	
Violations (mean±s.d.)[a]	
Distance constraints (Å)	0.12780 ± 0.00229
Dihedral angel constraints (°)	2.47094 ± 0.31544
Max. dihedral angle violation (°)	5
Max. distance constraint violation (Å)	0.5
Deviations from idealized geometry	
Bond lengths (Å)	0.00796 ± 0.00014
Bond angles (°)	0.94224 ± 0.01034
Impropers (°)	0.73831 ± 0.01966
Average pairwise r.m.s.d. (Å)	
Backbone atoms	0.584 ± 0.106
All heavy atoms	1.177 ± 0.097
Ramachandran Plot	
Residues in allowed region (%)	98.8
Residues in disallowed regions (%)	1.2

[a] Statistics were calculated over 20 structures with lowest energies.

Supplementary Table 2. Comparison of binding interface between Rap1 and its effector proteins[a,b,c]

	Rap1	F0	RIAM-RA PDB: 4DXA	KRIT1-F1 PDB: 4KVG	cRaf-1 PDB: 1GUA
α1 helix	S17	**K15**			
	V21	**K15**, I36		**R452**	
	V24	N12, V13			V88
Switch I	Q25	V13, I36, E38		**R452**	K87, V88, R89, G90
	I27	P37, E38			
	V29	P37			**K84**
	K31	E34, **R35**			**K84**
	D33	**K15**, **R35**	**K213**	**R432**, **R452**	**K84**
	P34	T16, **R35**		**R432**	
	I36	S5, T16, M17, Q18	V182, S194, L195, M196	S433, V434, E435	I57, V69, N71
β2 strand	E37	**K7**, T16	**K193**, S194	**R423**, Y431, S433	**R59**, R67, T68, V69
	D38	V14	T192, **K193**, S194	Y431, **R432**, S433	R67, T68, R89
	S39	V14	S191, T192	S430, Y431	Q66, R67, R89
	Y40		**K193**	**R452**	Q66, R89
	R41	N12, V13, V14	S190	D428, G429	N64, K65, Q66
	K42	N12			
β3 strand	M52				N64
	L56			Y431	
Switch II	T61	Q18			
	Q63	V2, Q18			
	F64	S5, **K7**, G76		F419	
	M67			K421	

[a] A cut-off of 4 Å was used to define the interface. The residues were identified in PyMOL 1.3 (Schrödinger, LLC.) and then manually organized into the table.

[b] Conserved residues are highlighted in red.

[c] The Rap1b/talin-F0 complex with the lowest energy was analyzed in this table.

Supplementary Table 3. Primer sequence information[a]

Plasmid construct	Primer sequence (5'-3')
pGST1-RIAM-RAPH (149-438)	Forward (EcoR I): ATTCCG**GAATTC**CCTGAACTTCTCTCTAAAGAAG<br>Reverse (Xho I): TATCCG**CTCGAG**TTATCCAGCTCTTGCCACAGCCC
pGST1-Rap1b	Forward (EcoR I): ATTCCG**GAATTC**ATGCGTGAGTATAAGCTTGTCG<br>Reverse (Xho I): TATCCG**CTCGAG**TTAAAGCAGCTGACCTGATGACTT
pET28t-Rap1b (1-167)	Forward (Nde I): GGCATTC**CATATG**CGTGAGTATAAGCTTGTCG<br>Reverse (Xho I): TATCCG**CTCGAG**TTATCTGTTGATTTGCCGCACTAG
talin (K15A, R35A)	Forward (K15A): TAGCATTGGGAATGTGGTGGCGACGATGCAATTTGAGCCA<br>Reverse (K15A): TGGCTCAAATTGCATCGTCGCCACCACATTCCCAATGCTA<br>Forward (R35A): GCATGATTCGTGAGGCGATCCCAGAGGCCC<br>Reverse (R35A): GGGCCTCTGGGATCGCCTCACGAATCATGC
Rap1b (G12V)	Forward: GTCGTTCTTGGCTCAGTAGGCGTTGGAAAGTCT<br>Reverse: AGACTTTCCAACGCCTACTGAGCCAAGAACGAC
Rap1b (I27H)	Forward: CAATTTGTTCAAGGACACTTTGTAGAAAAATACG<br>Reverse: CGTATTTTTCTACAAAGTGTCCTTGAACAAATTG
Rap1b (K31E)	Forward: GGAATTTTTGTAGAAGAGTACGATCCTACGATAG<br>Reverse: CTATCGTAGGATCGTACTCTTCTACAAAAATTCC
Rap1b (C51S, C118S, C141S)	Forward (C51S): GTAGATGCACAACAGAGTATGCTTGAAATCTTGG<br>Forward (C118S): CTTGTTGGTAATAAGAGTGACTTGGAAGATG<br>Forward (C141S): GCAAGACAATGGAACAACAGTGCATTCTTAGAATC

[a] Restriction sites are in bold and underlined.
